# The Effervescent Gallbladder: An Emergency Medicine Bedside Ultrasound Diagnosis of Emphysematous Cholecystitis

**DOI:** 10.7759/cureus.1520

**Published:** 2017-07-27

**Authors:** Brian B Wexler, Nova L Panebianco

**Affiliations:** 1 Department of Emergency Medicine, The Hospital of the University of Pennsylvania

**Keywords:** bedside ultrasound, abdominal pain

## Abstract

Emphysematous cholecystitis (EC) is a distinct clinical disease that carries a high rate of morbidity and mortality. Maintaining a high index of suspicion, especially in the right patient population, combined with emergency bedside ultrasound can lead to rapid diagnosis and initiation of treatment for this life threatening condition.

## Introduction

Abdominal pain and biliary diseases are common complaints and findings in emergency departments. Emphysematous cholecystitis (EC) is a rare but clinically important form of acute gallbladder inflammation separate from acute cholecystitis [1-3]. This rapidly progressive and potentially life threatening condition carries an associated mortality rate of 15%, is often insidious in onset, and may present with only vague abdominal complaints [2]. Knowledge of this condition and the sonographic findings of EC, coupled with appropriate clinical suspicion, is crucial to an expeditious diagnosis of this disease process.

We report a case where emergency medicine bedside ultrasound (EMBU) played a key role in the prompt diagnosis and initiation of appropriate treatment which led to further imaging and surgical consultation for this unique disease. Informed consent was obtained from the patient for this study.

## Case presentation

A 65-year-old male with a significant past medical history, including insulin dependent diabetes mellitus and nonischemic cardiomyopathy (status post left ventricular assist device placement) presented with a two-three days history of crampy abdominal pain, nausea, anorexia, and fever. The temperature on presentation was 103°F. A physical exam revealed an acute on chronically ill appearing male with mild right upper quadrant tenderness to palpation. Laboratory testing showed normal white blood cell count of 7.7 (thousand/mL), elevated alanine transaminase 91 (U/L), aspartate aminotransferase 199 (U/L), alkaline phosphatase 280 (U/L), total bilirubin 4.7 (mg/dL) and lactic acid 2.5 (mmol/L).

**Figure 1:**
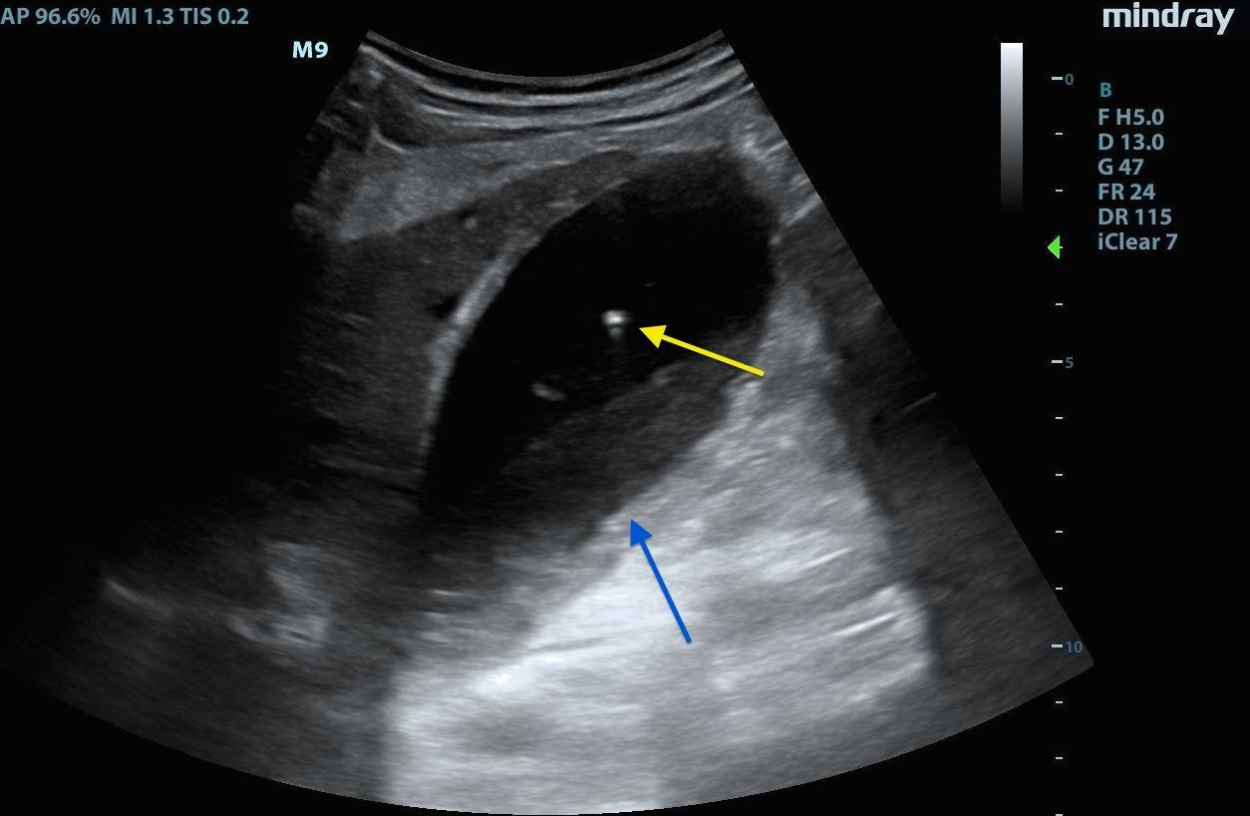
Image showing the gas rising in the gallbladder. Hyperechoic foci of gas (yellow arrow) rising non-dependently within gallbladder with sludge (blue arrow) and stones layering dependently.

**Figure 2:**
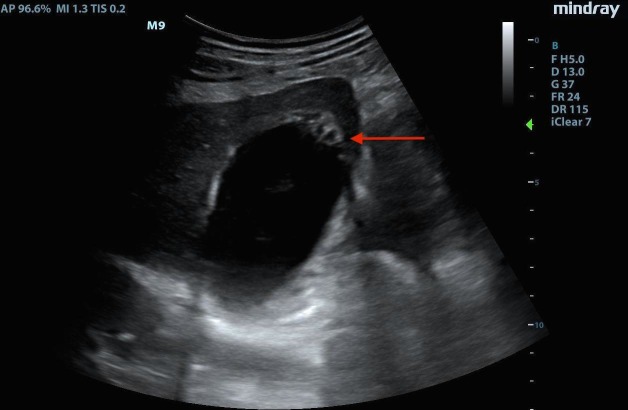
Image showing the thickened wall of the gallbladder. Thickened gallbladder wall with wall edema (red arrow).

EMBU of the right upper quadrant demonstrated a distended gallbladder with small layering gall stones, biliary sludge, a thickened gallbladder wall, small amounts of pericholecystic fluid, and most interestingly echogenic foci rising from the dependent part of the gallbladder towards the nondependent wall (gas). Refer below for sonographic findings (Figure 1-2).

**Figure 3:**
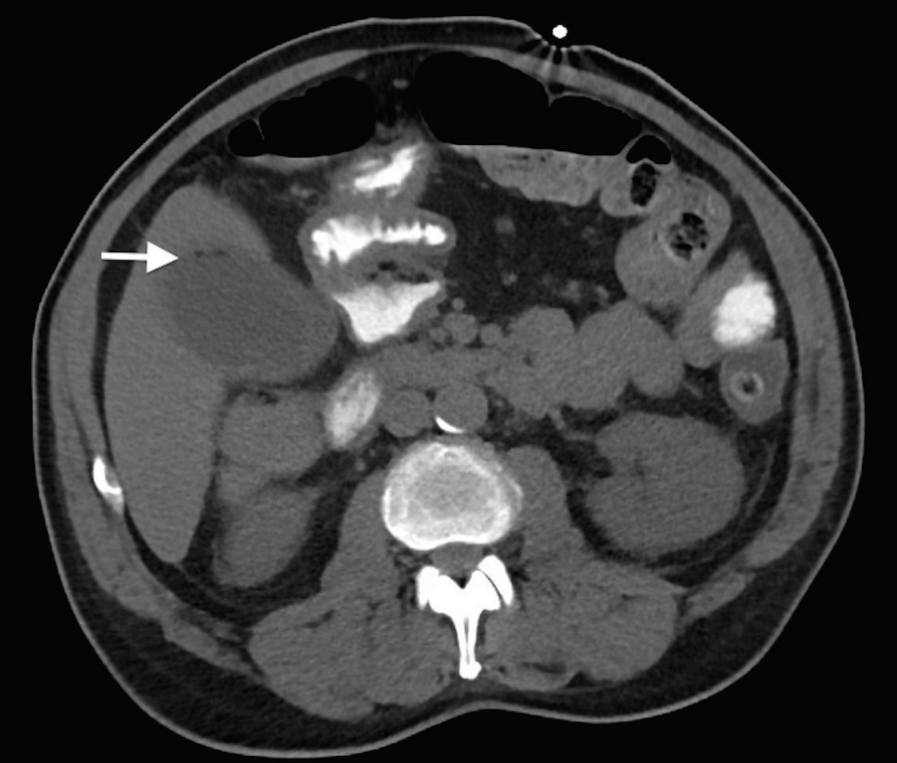
Image showing the computed tomography (CT) of the abdomen. Axial computed tomography (CT) image demonstrating collections of gas near the anterior gall bladder wall (white arrow).

Given the concern for EC from the above findings, computed tomography (CT) of the abdomen and a comprehensive radiology were performed and the right upper quadrant ultrasound was obtained. Abdominal CT showed gallbladder distension and wall thickening with a small amount of nondependent gas in the region of the gallbladder wall (Figure 3). The comprehensive radiology, ultrasound exam confirmed gallstones with a distended gallbladder, diffuse wall thickening with hyperemia, trace pericholecystic fluid as well as echogenic foci within the nondependent portion of the gallbladder lumen with shadowing concerning for gas. The patient was administered with Vancomycin and piperacillin/tazobactam, surgery was consulted for open cholecystectomy and admitted to the cardiac surgery intensive care unit.

During surgery, the gallbladder was noted to be thickened and edematous. The patient had an unremarkable intraoperative cholangiogram. Pathologic examination of the gallbladder revealed multiple flat black calculi, tan-red mucosa, and adherent yellow-brown material. The gallbladder wall ranged from 0.5-0.7 cm in thickness.

Blood cultures (obtained prior to administration of antibiotics) grew Enterobacter aerogenes and Clostridium perfringens. The patient was transitioned to oral levofloxacin and metronidazole and discharged from the hospital six days after surgery.

## Discussion

As stated above, the EC is a rare but distinct clinical disease. It occurs more commonly in males and diabetics and has high morbidity and mortality (15%) rates compared to acute cholecystitis despite its often deceptively mild clinical findings [1-2]. EC often progresses rapidly and carries five times higher risk of perforation. The etiology is thought to be related to vascular compromise of the cystic artery and its branches, leading to gallbladder ischemia, followed by gas forming organism proliferation which leads to penetration and accumulation of gas in the gallbladder and wall [2-3].

Classic sonographic findings of EC include highly echogenic collections of gas, with or without shadowing and reverberations, which originate from the gallbladder wall or lumen. These are often described as “powder snow like” speckled echoes. Another finding is termed the “effervescent gallbladder” in which gas bubbles are seen rising from the dependent portion of the gallbladder towards the nondependent portion. This has been described as similar to champagne bubbles rising in a glass [1-3].

It is important to consider other potential sources or causes of gas in the gallbladder/biliary tree in addition to gas forming infections. These include enterobiliary fistula or anastomosis, incompetent sphincter of Oddi, spontaneous internal biliary fistula, recent instrumentation such as endoscopic retrograde cholangiopancreatography (ERCP), medication side effects (including magnesium sulfate, atropine, nitroglycerin, and dopamine) and cholangitis [3].

Establishing the diagnosis of EC requires clinical suspicion and visualization of gas in the gallbladder lumen and/or wall. It is especially important to maintain a high index of suspicion when the gallbladder is poorly visualized, especially in patients with a history of diabetes. Treatment includes prompt cholecystectomy or if a patient is a poor surgical candidate, an alternative therapeutic option includes broad spectrum antibiotics in conjunction with percutaneous cholecystostomy [2, 4].

This case report emphasizes the role of EMBU in the rapid diagnosis of this concerning disease. Bedside images were obtained and interpreted within 20 minutes of the patient arriving at his emergency department room. Comparatively, the comprehensive right upper quadrant ultrasound by Radiology took 95 minutes from arrival time to obtain and 155 minutes from arrival for preliminary radiographic interpretation. CT images took 228 minutes from arrival to obtain and 351 minutes from arrival for preliminary radiographic interpretation.

## Conclusions

Given the high morbidity and mortality associated with emphysematous cholecystitis (EC), prompt diagnosis and appropriate management are crucial. An understanding of the sonographic findings of EC and use of emergency medicine bedside ultrasound (EMBU) can aid in the rapid identification of this distinct clinical entity.
